# Soil restoration with organic amendments: linking cellular functionality and ecosystem processes

**DOI:** 10.1038/srep15550

**Published:** 2015-10-27

**Authors:** F. Bastida, N. Selevsek, I. F. Torres, T. Hernández, C. García

**Affiliations:** 1 Department of Soil and Water Conservation, Centro de Edafología y Biología Aplicada del Segura, CEBAS-CSIC, Campus Universitario de Espinardo, Aptdo. de Correos 164, Espinardo 30100 Murcia, Spain; 2 Department of Agroforestry Technology and Science and Genetics, School of Advanced Agricultural Engineering, Castilla La Mancha University, Campus Universitario s/n, 02071 Albacete, Spain; 3 Functional Genomics Center Zurich, ETH Zurich/University of Zurich, Winterthurerstrasse 190, 8057 Zurich, Switzerland

## Abstract

A hot topic in recent decades, the application of organic amendments to arid-degraded soils has been shown to benefit microbially-mediated processes. However, despite the importance of soils for global sustainability, a gap has not been addressed yet in soil science: is there any connection between ecosystem-community processes, cellular functionality, and microbial lifestyles (i.e. oligotrophy-copiotrophy) in restored soils? Together with classical ecosystem indicators (fatty-acids, extracellular-enzyme activities, basal respiration), state-of-the-art metaproteomics was applied to fill this gap in a model-restoration experiment initiated 10-years ago by the addition of sewage-sludge and compost. Organic amendment strongly impacted ecosystem processes. Furthermore, the type of material used induced differences in the cellular functionalities through variations in the percentages of proteins involved in translation, transcription, energy production and C-fixation. We conclude that the long-term impact of organic restoration goes beyond ecosystem processes and affects cellular functionalities and phyla-lifestyles coupled with differences in microbial-community structures.

The misuse of soils and abandonment of agricultural areas after intensive utilization has led to the degradation of soils. Soil degradation processes are exacerbated under arid and semiarid climates, which affect nearly 4 × 10^9^ ha of the planet^1^. In these areas, water scarcity hinders plant growth, and the input of organic matter is therefore minimal. Under such nutrient limitation, the development and activity of the microbial community are highly compromised, as is the biogeochemical cycling of elements. These factors cause severe soil degradation and desertification^2^,^3^, as well as preventing soils from carrying out ecosystem functions and acting as substrates for agriculture.

Considering the human population growth and the threat of global change, the protection of soils as the main substrate for agriculture and ecosystem services is a mandatory duty. To fight soil degradation, restoration approaches have been proposed in order to recover soil fertility and improve soil quality. The application of organic wastes (i.e., sewage sludges, composts, pruning wastes, etc.) has been widely shown to be an adequate strategy for increasing the level of organic matter in soils, with benefits for the development of microorganisms^4^. Organic amendments usually foster plant development, and, together with the exogenous organic matter added with the amendments, such treatments improve the nutrient conditions of the soil and subsequent microbial growth^1^,^5^. Increases in microbial biomass are commonly linked to increases in the activity of the extracellular enzymes (linked to the C, N, and P cycles), which are able to degrade organic molecules into simpler compounds that are readily available for plant and microbial growth^6^,^7^.

The influence of organic amendments, both in the short- and long-term, on the structure and composition of the microbial community of arid and semiarid soils has also been explored using next-generation sequencing approaches^8^,^9^. For example, Chaudhry *et al.* (2012)^8^ found that *Proteobacteria*, *Bacteroidetes*, and *Gemmatimonadetes* were more abundant in organically-cultivated land than in a soil without organic inputs. Furthermore, Siles *et al.* (2014)^9^ suggested that certain bacterial groups (*Rhizobiales*, *Caulobacterales* and *Sphingomonadales*) benefited from amendments based on olive residues. However, these genomic approaches are not able to establish functional-phylogenetic relationships within the microbial community.

Proteomics has recently emerged as a potential tool for providing both functional and phylogenetic insights into complex microbial communities^10^, including soil samples^11^,^12^,^13^. Nevertheless, it is important to highlight that, currently, the intracellular information provided by metaproteomics is much more powerful than the identification of extracellular enzymes^11^,^13^,^14^. For this reason, the metabolic and cellular information provided by proteomics may be useful for tracking the responses that ultimately determine the lifestyles within the microbial community of soils subjected to organic restoration. In this vein, Fierer *et al.* (2007)^15^ proposed an ecological classification of bacterial phyla that separates them into “oligotrophic” and “copiotrophic” categories, depending on their metabolism and growth behaviour. Copiotrophs consume labile soil C sources and grow quickly when conditions are adequate. In contrast, oligotrophs grow more slowly and outcompete copiotrophs under low nutrient availability^15^,^16^. However, besides the importance of intracellular processes in determining the cellular functionality associated with lifestyles, these issues have not been elucidated so far. Proteomics may provide an added value, by linking cellular responses to phylogenetic variations in the soil community.

In this study, we have used a multidisciplinary methodological framework to explore the long-term responses of the microbial community to organic amendments (sewage sludge and compost obtained from it) in a well-established soil restoration field trial. By using phospholipid-fatty acids (PLFA), enzyme activities, and meta-proteomics, we aim to reveal the relationships between community structure and biomass, functionality, and lifestyles of the dominant microbial groups. Organic amendments usually increase the microbial biomass and the extracellular enzyme activities of degraded soils^6^,^7^, as well as altering the structure of the microbial communities^8^,^9^. In contrast, the intracellular responses of the microbial communities to soil restoration are less well-understood. We hypothesized that the type of organic amendment would not impact the intracellular and metabolic processes (i.e. cell cycle control, carbohydrate and amino acid metabolism, energy production, etc.) or the lifestyles of microbial groups that probably are more conserved along lineages.

## Results

### Plant cover, and the activity and PLFA content of the microbial community

Plant cover was higher in restored plots (81% in compost-amended plots and 85% in sludge-amended plots) than in control plots (42%), indicative of successful soil restoration. The contents of organic C and total N were significantly higher in the restored plots than in the controls, with compost-treated plots showing the highest values (P < 0.05) (Table 1). The content of water-soluble C (WSC) was also higher in the restored plots, being higher in sludge-amended than in compost-amended plots. The C/N ratio was lowest in the sludge-amended plots (P < 0.05) (Table 1).

All enzyme activities were higher in amended plots than in control plots (P < 0.05) (Table 1). With the exception of polyphenol oxidase, enzyme activities were highest in the compost treatments. Soil respiration was higher in amended than in control plots, but no significant differences were observed between compost- and sludge-amended plots.

**Figure 1:**
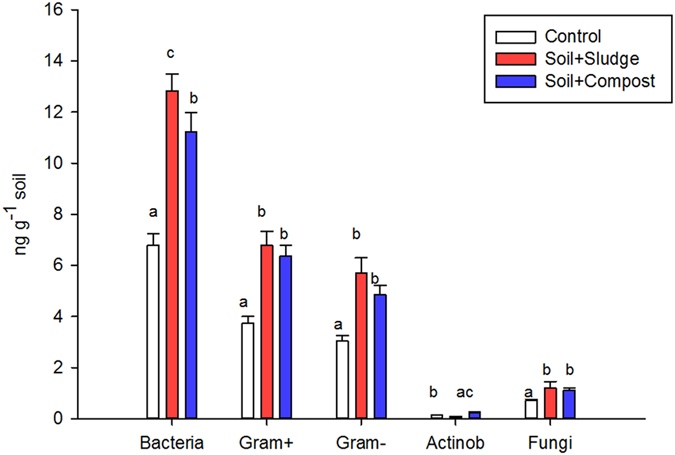
PLFA content of different microbial groups in control and restored plots. Data followed by the same letter are not significantly different according to the HSD test (P < 0.05). Actinob = Actinobacteria.

The PLFA content of the different microbial groups was higher in the amended plots than in the control plots (P < 0.05). The bacterial PLFA content was higher in the sludge-amended plots than in the compost-treated plots (P < 0.05) (Fig. 1). However, the contents of Gram-positive and Gram-negative fatty acids were not differentially affected by the sludge and compost amendments.

### Protein-based phylogenetic composition of the microbial community

A total of 10818 redundant proteins were identified, which were classified into 1351 protein groups. The amount of bacterial proteins was significantly greater (up to 20-times) than that of fungal proteins. Moreover, the percentage of bacterial proteins was significantly higher in the amended plots than in the control ones (P < 0.05), with no differences between compost- and sludge-treated soils (Table 2). Accordingly, the percentage ratio of fungal-to-bacterial proteins was significantly lower in the amended plots than in control ones (P < 0.05). In terms of fungal origin, the abundance of *Ascomycota* (up to 96% of the fungal proteins) was higher than the abundance of *Basidiomycota* (up to 14%) in all treatments. Moreover, the abundance of proteins from *Ascomycota* was higher in the control (94%) and compost plots (96%) than in the sludge treatments (86%) (Table 2).

**Figure 2:**
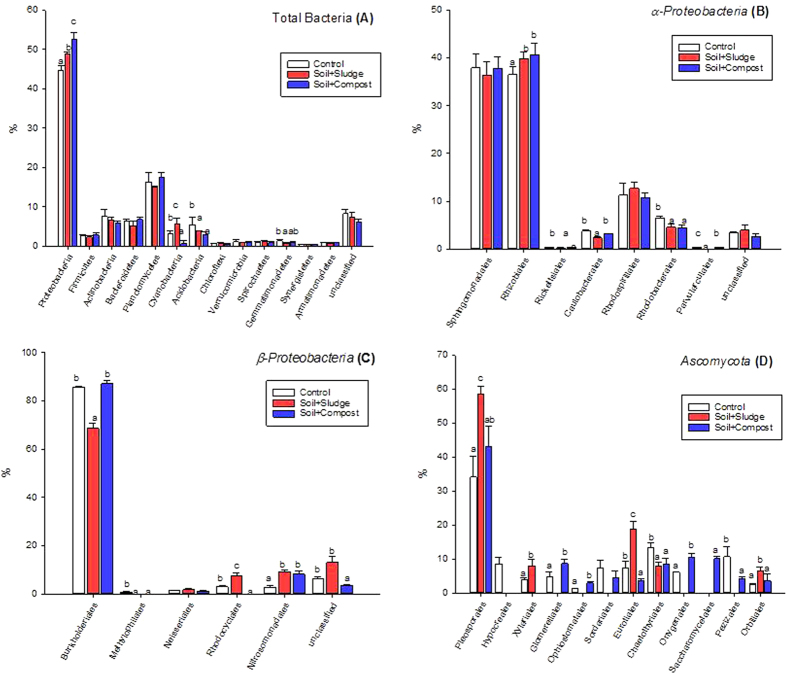
The distribution of total bacterial (A), α-Proteobacterial (B), β-Proteobacterial (C) and Ascomycotal (D) proteins in control and restored plots. Data followed by the same letter are not significantly different according to the HSD test (P < 0.05).

With regard to protein abundances, the bacterial communities of control, compost-, and sludge-amended plots were dominated by *Proteobacteria*, *Planctomycetes*, *Actinobacteria*, *Bacteroidetes, Cyanobacteria, Firmicutes* and *Acidobacteria* (Fig. 2A). Among these phyla, *Proteobacteria* and *Planctomycetes* represented more than 60% of the proteins. The composition of the bacterial community only differed between treatments in the case of *Proteobacteria*, *Cyanobacteria*, *Acidobacteria* and *Gemmatimonadetes* (Fig. 2A). The percentage of cyanobacterial proteins was lowest in compost-amended plots (0.6%) and was higher in the sludge treatment (6%) than in the control plots (3%). The percentage of acidobacterial proteins decreased with the addition of organic amendments (P < 0.05). The proteobacterial-to-acidobacterial protein ratio was higher in compost plots (21.50) than in sludge-treated plots (12.41) and was lowest in control plots (7.28) (P < 0.05).

The *α-Proteobacteria* and *β-Proteobacteria* represented the dominant proteobacterial classes, with almost 80% of the proteobacterial proteins. In the case of *α-Proteobacteria*, the percentage of proteins stemming from *Rhizobiales* increased significantly with soil restoration (P < 0.05) (Fig. 2B). In contrast, the percentage of proteins from *Rhodospirillales* was higher in the control than in the amended plots (P < 0.05). A detailed analysis of *β-proteobacteria* orders revealed a lower abundance of *Burkholderiales* proteins in sludge-treated plots (69%) than in the other treatments (around 86%), as well as a higher percentage of *Nitrosomonadales* proteins in amended plots (8.2% and 9.2% in compost- and sludge-treated plots, respectively) in comparison to control plots (2.8%) (P < 0.05) (Fig. 2C).

Considering the dominance of *Ascomycota* within the fungal community, the phylogenetic composition of this group was explored in detail. *Ascomycota* was dominated by *Pleosporales*, with up to 60% of the proteins (Fig. 2D). The percentages of proteins from *Pleosporales*, *Xylariales*, *Eurotiales* and *Orbiliales* increased significantly with sludge amendment, in comparison to control plots (Fig. 2D). In contrast, proteins from *Glomerellales*, *Sordariales*, *Ophistomatatales*, *Onygenales* and *Pezizales* were not identified in sludge-treated plots. The percentages of proteins from *Glomerellales*, *Saccharomycetales*, *Pezizales*, *Ophistomatatales* and *Onygenales* increased significantly with compost amendment, in comparison to control plots (P < 0.05). Regardless of the type of organic amendment, soil restoration decreased the abundances of *Hypocreales* and *Chaetothyriales* proteins. Indeed, *Hypocreales* proteins were not detected in amended plots (Fig. 2D).

**Figure 3:**
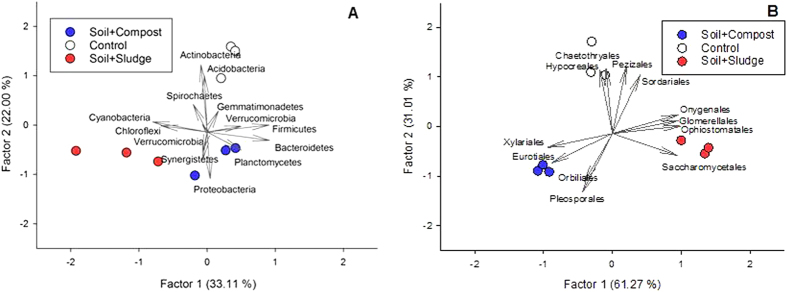
Factor analysis of protein-based phylogeny, illustrating changes in the structure of the bacterial (**A**) and fungal (**B**) communities.

The structure of the bacterial community was analyzed by factor analysis of the proteins categorised at the phylum level. Factor 1 explained 33.11% of the variance and Factor 2 explained 22.00%. With respect to Factor 1, the bacterial community structures of control and compost-treated plots grouped together, separated from sludge-amended plots (P < 0.05) (Fig. 3A). *Firmicutes*, *Bacteroidetes*, *Chloroflexi* and *Cyanobacteria* received the highest loading scores in Factor 1. This factor separated sludge amended plots from the other treatments. *Actinobacteria*, *Acidobacteria* and *Proteobacteria* received the highest loading scores in Factor 2. This factor separated amended plots from control plots (P < 0.05) (Fig. 3A).

The structure of the fungal community was analyzed by factor analysis of the proteins categorised at the order level. Factor 1 explained 61.27% of the variance and Factor 2 explained 31.01%. With respect to Factor 1, the fungal community structures of control, sludge- and compost-treated plots were different (P < 0.05) (Fig. 3B). *Xylariales*, *Eurotiales*, *Onygenales*, *Glomerellales*, and *Ophiostomatales* explained differences between the control, sludge- and compost-treated plots. Furthermore, considering Factor 2, the structure of the fungal community of restored plots was different to that of control plots. These differences were explained by *Pleosporales*, *Orbiliales*, *Sordariales*, *Pezizales*, *Chaetothryales* and *Hypocreales* (Fig. 3B).

The Shannon and Simpson indexes of bacterial and fungal diversity were significantly higher in the control plots than in the restored plots (P < 0.05) (Table 2). Moreover, the indexes of bacterial diversity were higher in the sludge treatments than in compost-amended plots (P < 0.05).

### Microbial functionality and lifestyles retrieved by proteomics

**Figure 4:**
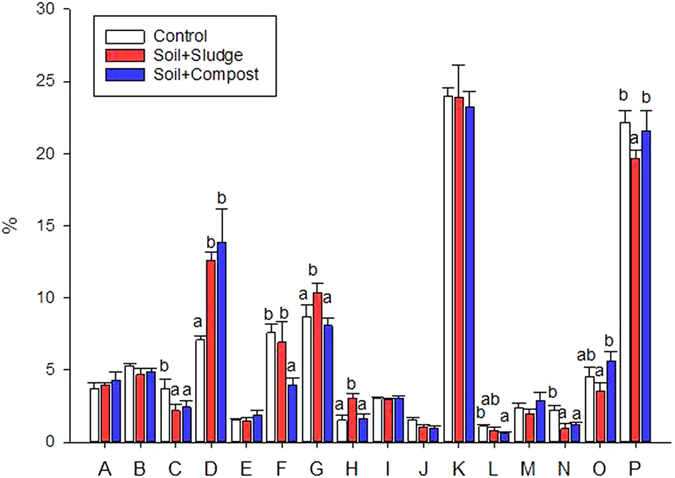
Functional classification of microbial proteins in control and restored plots. Data followed by the same letter are not significantly different according to the HSD test (P < 0.05). Abbreviations: A (Amino acid transport and metabolism); B (Carbohydrate transport and metabolism); C (Cell cycle control, cell division, chromosome partitioning); D (Cell wall, membrane, envelope biogenesis), E (Coenzyme transport and metabolism); F (Cytoskeleton); G (Energy production and conversion); H (Function unknown); I (Inorganic ion transport and metabolism); J (Nucleotide transport and metabolism); K (Posttranslational modification, protein turnover, chaperones); L (Replication, recombination and repair); M (Secondary metabolites biosynthesis, transport and catabolism); N (Signal transduction mechanisms); O (Transcription); and P (Translation, ribosomal structure and biogenesis).

Overall, the categorisation of bacterial and fungal proteins by their functional role revealed that a high amount of proteins were devoted to “Posttranslational modification, protein turnover and chaperones” (up to 25%); “Translation, ribosomal structure and biogenesis” (up to 22%); “Cell wall/membrane/envelope biogenesis” (up to 14%); and “Energy production and conversion” (up to 10%) (Fig. 4).

The percentage of proteins involved in “Cell wall/membrane/envelope biogenesis” was higher in the amended plots (approximately 13%) in comparison to the control (7%) (P < 0.05). Furthermore, the relative abundance of proteins involved in “Energy production and conversion” was higher in the sludge-amended plots (10.3%) than in the compost (8.0%) and control plots (8.6%) (P < 0.05) (Fig. 4).

**Figure 5:**
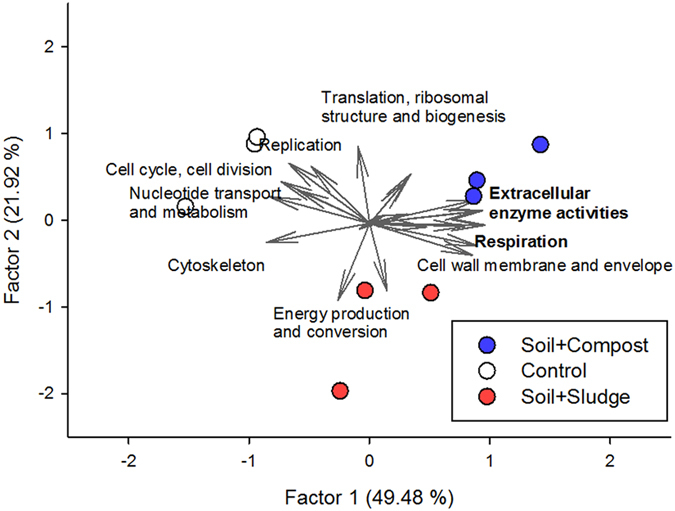
Factor analysis of microbial protein-functional groups and enzyme activities, illustrating changes in the structure of the microbial community.

In order to look at relationships between the biogeochemical processes mediated by extracellular enzymes and metabolic-intracellular processes in the different samples, a factor analysis of the relative abundance of each functional protein group, including both bacterial and fungal proteins, and the extracellular enzyme activities was performed (Fig. 5). Factor 1 explained 49.48% of the variance and Factor 2 explained 21.92%. The functional structures of control and sludge- and compost-amended plots differed in terms of Factor 1 (P < 0.05).

Within Factor 1, extracellular enzymes (β-glucosidase, cellulase, lipase, phosphatase and urease) and proteins related to “Cell wall/membrane/envelope biogenesis” and “Nucleotide transport and metabolism” explained differences between control, sludge- and compost-treated plots. Factor 2 separated the sludge plots from the control and compost plots. Proteins involved in the following intracellular processes (“Energy production and conversion”; “Signal transduction mechanisms”; “Carbohydrate transport and metabolism”; “Replication” and “Translation, ribosomal structure and biogenesis”) explained differences between sludge-treated plots and the other treatments.

**Figure 6:**
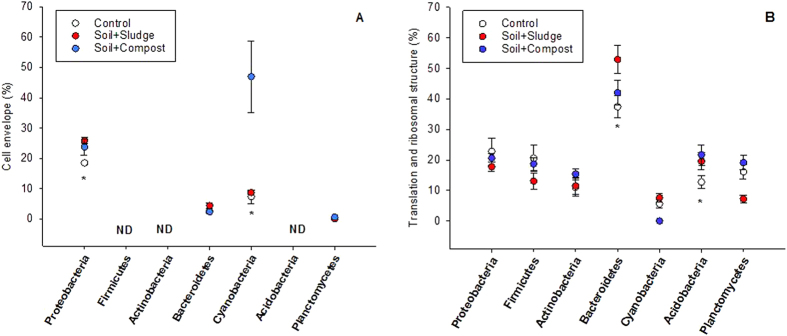
Cell wall and envelope proteins (A) and Translation and ribosomal proteins (B). Data are expressed as the percentage of the total amount of proteins of each phylum and treatment. ND = not detected. * indicates significant differences (P < 0.05) between control and restoration treatments.

The percentages of “cell wall membrane and envelope” proteins and “translation and ribosomal” proteins were taken as functional indicators of copiotrophy (Fig. 6). It is important to highlight the absence of specific biomass proteins from *Firmicutes*, *Actinobacteria* and *Acidobacteria*. Conversely, *Proteobacteria* and *Cyanobacteria* showed higher values of the “cell wall membranes and envelope” proteins than the other phyla. Moreover, in the case of *Proteobacteria*, the percentage of “cell wall membranes and envelope” proteins was greater in the restored plots than in control plots. However, in the case of *Cyanobacteria*, only compost-amended plots showed a higher percentage in comparison to control plots (P < 0.05) (Fig. 6A). The percentage of “translation and ribosomal” proteins was highest in *Bacteroidetes* (Fig. 6B). This percentage was higher in restored plots than in control plots only for *Bacteroidetes* and *Acidobacteria*.

The amount of cyanobacterial proteins involved in C-fixation (ribulose bisphosphate carboxylase and phycocyanins), as calculated by the NSAF values, was highest in sludge-treated plots (0.037), in comparison to compost-restored plots (no C-fixing proteins) and control plots (0.013) (Table S2).

### Microbial growth with respect to energy processes

Proteomics was used to obtain indicators of bacterial and fungal growth with respect to energy processes (BG-En and FG-En, respectively) (Table 2). BG-En and FG-En were not significantly different in control and compost-amended plots. However, FG-En was higher than BG-En in the sludge-amended plots. BG-En was higher in compost than in sludge-amended plots (P < 0.05), while FG-En was highest in sludge-amended plots.

## Discussion

The responses of soil microbial communities to organic amendments were investigated at cellular and ecosystem levels by several approaches that include phospholipid fatty acids, extracellular-enzyme activities and metaproteomics. Our results provide novel knowledge about the cellular and phylogenetic responses of soil microbial populations to long-term restoration under semiarid climate.

The higher levels of nutrients, organic matter and enzyme activities in restored soils were related to a greater microbial biomass in comparison to the control plots, as has been observed also by other authors^6^,^17^. However, the superior organic C and N contents and enzyme activities in compost-treated soils did not always translate into a greater microbial biomass with respect to the sludge-treated plots. Moreover, despite the absence of differences in soil respiration between the two amendment treatments, the total bacterial PLFA content was higher in sludge-treated plots, although the sludge and compost treatments did not significantly differ in their Gram-positive and Gram-negative fatty acid contents. All together, these results point to a decoupling of nutrient content and microbial activity on the one hand and the generation of microbial biomass on the other. Recently, Lee & Schmidt (2014)^18^ also indicated that microbial activity and microbial biomass are not always directly linked and suggested that the efficiency of microbial growth can partially explain this phenomenon. In this sense, through the use of metaproteomics, we have been able to provide different lines of evidence that question some basic assumptions of soil microbial ecology, which has traditionally supposed higher fungal than bacterial growth efficiency and a positive relationship between fungal growth efficiency and the C/N ratio^19^,^20^. We quantified the ratio between the total proteome and energy production proteins (mainly ATPase) in the bacterial and fungal communities, separately. From these calculations, two major findings can be highlighted. First of all, despite the fact that the lowest C/N ratio was found in sludge-amended soils, FG-En, as evaluated by proteomics, was highest in these same soils. Our results therefore do not support a positive relationship between C/N and fungal growth efficiency. Secondly, with the exception of sludge-amended plots, FG-En was not higher than BG-En. This result agrees with the observations of Thiet *et al.* (2006)^21^, who rejected the fact that soil fungi have greater growth efficiency than bacteria. Interestingly, Bradford *et al.* (2013)^22^ indicated that an increase in the growth efficiency of the bacterial community was related to the formation of soil organic matter. Our results showed that compost, the treatment with the greatest TOC content, also showed higher BG-En than sludge-treated plots.

Genomic approaches have revealed an impact of organic amendments on the structure and composition of soil microbial communities^23^,^24^ and some researchers^25^,^26^ – through the use of enzyme activities and community-level physiological profiles - suggested a relationship between phylogenetic and functional processes. Interestingly, by using metaproteomics, we were able to link changes in the community structure at the phylogenetic and functional levels. A detailed examination of the factor analysis, including protein-functional groups and extracellular enzyme activities, revealed two findings. Firstly, the main differences between the restored and non-restored plots are related to: i) the level of microbial biomass and the extracellular enzyme activities (which were higher in the restored than in control plots), and ii) the amounts of “cell cycle and cell division” and “replication” proteins (which were higher in control plots). These results suggest an intense turnover of the biomass that does not sustain microbial growth when nutrient resources are limited (control plots). Secondly, the type of amendment influences the intracellular metabolism and associated lifestyles of the bacterial community inhabiting restored soils. These differences can be mediated by changes in the structure of the bacterial and fungal communities, as showed by factor analysis. The stabilized nature of compost in comparison to sludge and the different plant communities promoted by each material after amendment^17^, could promote microbial communities with different physiology. For the first time, differences in the cellular metabolic functioning of the soil microbial community in response to different organic amendments have been highlighted.

In parallel with the functional and phylogenetic changes, the bacterial and fungal communities subjected to restoration showed less diversity than in non-restored plots. Recently, several studies have observed a negative correlation between plant cover and bacterial diversity^26^. It is plausible that the greater plant cover in restored plots provided cellulose and lignin inputs to the soil that require a “more specialised” (and less diverse) community for their processing. However, it is worthy of note that microbial diversity was not related to ecosystemic processes. Indeed, basal respiration and the activity of extracellular enzymes were higher in restored plots even though these plots showed lower diversity than the control. This finding supports the idea that changes in community composition rather than in diversity are of the greatest importance for the cycling of elements in soil^27^.

The bacterial community was dominated by *Proteobacteria*, as reported by other researchers in arid areas^28^,^29^. The proteobacterial community increased significantly in the restored soils, particularly in compost-treated plots. Restored soil presented a greater amount of plant cover and, as is typical of copiotrophic organisms, *Proteobacteria* have been observed to respond positively to the presence of vegetation and nutrient improvements^30^. Indeed, positive and significant correlation coefficients were observed between the proteobacterial community and TOC or total N. Nevertheless, the abundance of *Proteobacteria* was higher in compost-treated plots than in sludge-treated plots. The chemical composition of compost (a more stabilized material) in comparison to sludge, as well as the initial differences in the plant community^17^ could impact the development of *Proteobacteria.* In accordance with their copiotrophic character, *Proteobacteria* are considered to have a high growth rate when conditions are adequate^15^,^31^. In agreement with this growth efficiency, the percentage of proteins assigned to “cell wall membrane and envelope” (an indicator of microbial biomass) was higher in *Proteobacteria* than in the rest of the populations in control plots. Moreover, the percentage increased with soil restoration - related to the higher availability of nutrients. Within the *α-Proteobacteria*, *Rhizobiales* proteins represented around 40% of the proteins. *Rhizobiales* is an N-fixing order of soil bacteria common in the rhizosphere and its abundance increased with organic amendment. This finding was expected since the vegetation cover was also greater in the amended plots than in the control plots and plant development after organic amendment is fundamental for soil restoration in arid ecosystems^25^,^32^. Moreover, it is also worth mentioning that the percentage of *Nitrosomonadales* was greater in restored plots than in control plots. This order is involved in the bottleneck of nitrification: the transformation of NH_4_^+^ to NO_3_^−^33. Previously, Bastida *et al.* (2009)^34^ demonstrated that organic amendments increase the copy number of genes involved in nitrification.

In contrast, although *Bacteroidetes* have been proposed as copiotrophic^15^, we found no statistical correlation between the abundance of this group and nutrient contents. However, proteomics point to a potential capacity for copiotrophy which, finally, is not phenotypically linked to a high biomass or consistent responses to nutrient improvement in restored plots. In brief, Klappenbach *et al.* (2000)^35^ proposed that an increased copy number of the rRNA operon is related to ecological strategies of bacteria for exploitation of nutrients. However, the higher amount of translation and ribosomal proteins in *Bacteroidetes* (particularly in restored plots) was not reflected in an increased biomass. It could be argued that *Proteobacteria* are superior competitors that exclude other bacterial lineages that *a priori* should have shown the same copiotrophic potential^29^,^36^.

*Acidobacteria* represented a phylum with low abundance in this study (in comparison to *Proteobacteria* or *Planctomycetes*). The abundance of this bacterial phylum is greatly governed by pH^37^ and the studied soil, with a pH > 7.5, did not seem to be the best habitat for *Acidobacteria*. Regarding lifestyles, *Acidobacteria* have usually been proposed as oligotrophic organisms^15^. We agree with this assumption, since the abundance of this phylum has been correlated negatively with nutrient levels^31^,^38^,^39^ and did not increase with restoration. Moreover, specific cell wall membrane proteins from this group were not found. Some studies have proposed that the ratio between *Proteobacteria* and *Acidobacteria* reflects the trophic status of the soil, with greater ratios found in copiotrophic environments with high nutrient availability^38^. Our proteome-based phylogeny suggests an overall copiotrophic pattern in the microbial community, linked to soil restoration (compost > sludge > control). It is striking that this pattern is prominent even 10 years after the one-time application of organic amendments, and might indicate a continuous evolution in the metabolic processes of the soil microbial communities.

As in the case of *Acidobacteria*, membrane cell wall and envelope proteins from *Firmicutes* and *Actinobacteria* were not identified, indicating an oligotrophic lifestyle for these phyla. Fierer *et al.* (2007)^15^ proposed that *Actinobacteria* and *Firmicutes* could not be assigned to any of the groups in an ecological gradient in the United States. Similarly, the cell wall membrane and envelope proteins were also of very low adundance in the case of *Planctomycetes* and this finding suggests an oligotrophic lifestyle for this unknown^40^, yet dominant (18%) phylum.

*Cyanobacteria* represented up to 5.75% of the bacterial community. Their relative abundance was lowest in the compost-amended plots, which showed the highest TOC content. Several studies have reported an increased abundance of *Cyanobacteria* in soils without vegetation or of low organic C content^13^,^28^. *Cyanobacteria* exhibited a high percentage of cell wall membrane and envelope proteins in compost-treated plots but this feature did not seem to be linked to CO_2_-fixation processes. However, soil restoration with sludge induced a sustainable, long-term increase in the abundance of *Cyanobacteria*, which was coupled to the identification of proteins involved in C-fixation. Considering the increase in the relative abundance of this phylum in the sludge-amended plots and the capacity of these bacteria for C-fixation, further research is needed on the possibilities of enhanced C-fixation in sludge-restored soils.

The reduced size of the fungal genome database probably limited the identification of fungal proteins, in comparison to bacterial ones, at the functional level. Nevertheless, phylogenetic information could be retrieved and revealed that soil restoration affected the structure of the fungal community, as in the case of the bacterial community. Moreover, the type of amendment (compost vs sludge) impacted the structure of the fungal community.

The effects of organic amendments on the fungal community have not been widely explored. Organic wastes have been shown to alter the structure of the fungal community in the long-term^25^, as described here. Furthermore, Siles *et al.* (2014)^9^, by pyrosequencing of the 28S-rRNA gene, found that the structure of the fungal community was affected by the application of olive residues in the short-term. However, our results do not match those found by Siles *et al.* (2014)^9^ in terms of composition of the fungal community. For instance, *Pleosporales* (the most-abundant fungal group) benefited from the sludge application, in comparison to the control, and showed a positive correlation coefficient with TOC in the long-term. However, Siles *et al.* (2014)^9^ found a reduced abundance of this group after the application of olive residues in the short-term (60 days)^9^. The response of other fungal groups depended on the type of organic amendment. For instance, *Glomerellales*, *Onygenales*, and *Saccharomycetales* showed higher abundances in compost-treated plots than in sludge plots. As mentioned above, chemical differences between the added sludge or compost and variations in the vegetal community fostered by each material could influence selectively the development of some fungal populations.

Metaproteomics revealed that the well-established improvement of ecosystem processes after organic restoration (i.e. biogeochemical cycling and biomass) and the associated changes in the phylogenetic structure of the bacterial and fungal communities are parallel to a decrease in microbial diversity and change in the functionality at the cellular level. With the exception of proteins related to biomass, the response of the cellular functionality did not follow the same pattern as the improvement of the ecosystem processes with soil restoration, which points to a disconnection of the extracellular and cellular environments.

At the functional level, differences between restored and non-restored soils are mainly controlled by extracellular enzyme activity and proteins linked to microbial biomass. Surprisingly, in the long-term, the type of organic amendment (sludge vs compost) influenced the functional structure of the microbial community and proteins involved in energy production; translation-ribosomal proteins are deeply involved in such differences.

Proteomics showed a domination of *Proteobacteria* in the microbial community and a copiotrophic behavior in response to organic amendments. In contrast, this study has questioned the copiotrophic capacity of *Bacteroidetes* in response to organic restoration. *Cyanobacteria* exhibited high sensitivity to the type of organic amendment and a molecular predisposition to C-fixation in sludge-amended plots.

## Methods

### Study area and experimental design

The experimental plots were located in Murcia (southeast Spain), in an area greatly affected by soil degradation processes. The climate is semiarid Mediterranean. The mean annual rainfall is 300 mm, and the rainfall distribution throughout the year is very irregular with two maxima (in October and April). The mean annual temperature is 17 °C. The studied soil has a sandy clay loam texture and is classified as an Aridic calcisol^41^. The vegetation of the study area is open Mediterranean scrub with species such as *Asphodelus fistulosus*, *Salsola genistoides*, *Piptatherum miliaceum*, *Dactylis* sp., *Rosmarinus officinalis* and *Stipa capensis*.

On March 25, 2004, nine 4 m × 5 m plots were established randomly in the experimental area (38°1′N 1°12′W). Each plot was separated from the others by a corridor (2 m wide). This area was almost bare, without vegetation, and was abandoned 10 years before, in 1994. Soil abandonment, inadequate climate conditions and the loss of plant cover are considered as the main initiators of soil degradation and desertification in arid areas. In order to fight against soil degradation, in three of the plots, sewage sludge from a water-treatment plant located in Murcia was added at a rate of 12 kg m^−2^. The sludge had been anaerobically digested for stabilisation and hygienisation. Compost made from the same material, with straw as bulking material, was added to another three plots (CM) at 12 kg m^−2^. The remaining three plots received no amendment and acted as controls. The compost and sewage sludge were incorporated into the top 15 cm of the soil using a rotovator. The control plots were also subjected to rotovator treatment. The plots were left in natural conditions.

After 10 years, in April 2014, the plots were sampled. The samples were sieved (at <2 mm) and stored at 4 °C for two weeks until biochemical analysis and at −20 °C until proteomic analysis. Before sieving, all debris and plant remains were removed in order to avoid any influence on the parameters analysed. In each plot eight subsamples were collected and pooled together in a composite soil sample, giving one biological replicate for each plot. Samples were collected from the upper 15 cm of the surface soil with hand-driven probes (10 cm in diameter). Analytical characteristics of the organic amendments are presented in the Supplementary Information (Table S1, Supplementary).

### Chemical analyses, basal respiration, enzyme activities and PLFA analysis

The pH was measured in a 1/5 (w/v) aqueous soil extract, in a pH meter (Crison mod.2001, Barcelona, Spain). Total organic carbon (TOC) was determined using a C analyser (Thermo Finnigan Flash EA 1112). Water-soluble carbon (WSC) was determined through soil extraction (2 h of shaking; soil:distilled water ratio of 1:5), followed by centrifugation, filtration and analysis of the extract solution in a C analyser for liquid samples (Shimazdu 5050A).

Microbial respiration (CO_2_ emission) was measured in 10-ml capped tubes containing 1 g of soil. Soil samples were moistened with distilled water to 60% of their water-holding capacity. The vials were then closed hermetically and incubated in the dark at 28 °C for 11 days. The concentration of CO_2_ was analysed periodically with a gas chromatograph (Trace Ultra Thermo Scientific, Milan (Italy)), using a packed column (Trace PLOT TG-BOND Q GC, Trace Ultra Thermo Scientific, Milan (Italy)).

Urease, phosphomonoesterase and β-glucosidase activities were determined by conventional methods, as reported by Bastida *et al.* (2014)^42^ (Supplementary information). Polyphenol oxidase was determined by the method of Allison (2006)^43^. Lipase was measured according to the method of Margesin *et al.* (2002)^44^. Cellulase activity was determined by following a modification of the method of Deng & Tabatabai (1994)^45^.

Phospholipids were extracted from 6 g of soil using chloroform-methanol extraction, as described by Bligh & Dyer (1959)^46^, and were fractionated and quantified using the procedure described by Frostegard *et al.* (1993)^47^. A detailed description of the PLFA methodology is provided in the Supplementary Information.

### Protein extraction and sample preparation for mass spectrometry analysis

Protein extraction was performed according to the method described by Chourey *et al.* (2010)^11^, which was tested beforehand for semiarid soils (Bastida *et al.*, 2014)^13^. The cell lysis and disruption of soil aggregates were performed by boiling for 10 minutes in SDS-buffer. The concentration and purification steps were performed using trichloroacetic acid (TCA) and three acetone washing steps. The protein content to be loaded in gels was equalized by the quantification of amino acids by liquid chromatography^13^ (Supplementary Information). Protein pellets were resuspended in sodium dodecyl sulphate (SDS) lysis buffer, containing 4% SDS, 0.1 mM dithiothreitol and 100 mM Tris HCl, and incubated for 5 min at 95 °C. High-intensity focused ultrasound (HIFU) was performed to solubilise proteins. Protein extracts were then subjected to ultrafiltration for detergent removal, cysteine alkylation and protein digestion according to Wisniewski *et al.* (2009)^48^. After separation by nano-HPLC, tryptic peptides were analysed on a Q Exactive mass spectrometer (Thermo Fisher Scientific, Bremen, Germany). Further details are available in the Supplementary Information.

### Database searching and bio-informatic classification of protein groups

Each file was searched with Mascot (version 2.4.1) against the NCBInr database (08/24/2014, containing 48,094,830 sequences). For the search, one missed cleavage per peptide was allowed, and we used carbamidomethylation as a fixed modification on cysteine residues and oxidation as a variable modification on methionine residues. Searches were performed with a parent-ion mass tolerance of ±10 ppm and a fragment-ion mass tolerance of ±0.05 Da. The Mascot search results were imported to Scaffold (version Scaffold 4, Proteome Software, Portland, OR, USA) to validate the MS/MS-based peptide and protein identifications. Proteins were considered to be identified with a protein threshold of 90% and a minimum of two peptides.

The “PROteomics results Pruning & Homology group ANotation Engine” (PROPHANE)^49^ ( http://www.prophane.de/index.php?p=new) was used to assign proteins to their phylogenetic and functional origin. Protein abundances were calculated based on the normalised spectral abundance factor (NSAF)^50^. The diversity of bacterial communities was calculated at the phylum and order level, respectively, according to the Shannon and Simpson indexes. In order to obtain an indicator of bacterial growth with respect to energy processes (BG-En), the sum of the NSAF values of the total bacterial proteins was divided by the sum of the NSAF values of bacterial proteins involved in energy production (mainly F0F1 ATP synthase). The fungal growth with respect to energy processes (FG-En) was obtained in a similar manner, but using only fungal proteins.

### Statistical analysis

Statistical analyses were performed using IBM-SPSS Statistics (version 19.0) software. In order to determine pair-wise differences between the treatments, the data were analysed using one-way ANOVA followed by the Tukey post-hoc test (HSD, P < 0.05). The structure of the microbial community was visualised using multivariate factor analysis, with the relative abundances of phyla based on proteomics and the relative abundances of protein functional groups and enzyme activities. Non-parametric multivariate analysis of ANOVA was performed on the groups obtained by factor analysis.

## Additional Information

**How to cite this article**: Bastida, F. *et al.* Soil restoration with organic amendments: linking cellular functionality and ecosystem processes. *Sci. Rep.* **5**, 15550; doi: 10.1038/srep15550 (2015).

## Supplementary Material

Supplementary Information

## Figures and Tables

**Table 1:** Chemical properties, microbial respiration and enzyme activities in control and restored plots.

	Control		Soil + Sludge		Soil + Compost	
	*Mean*	*Std*	*Mean*	*Std*	*Mean*	*Std*
pH	7.47 a	0.21	7.64 a	0.15	7.59 a	0.88
Total Organic C (g 100g^−1^)	2.07 a	0.21	3.06 b	0.24	5.66 c	0.61
Total N (g 100g^−1^)	0.16 a	0.02	0.32 b	0.02	0.49 c	0.03
C/N ratio	13.32 b	1.64	9.64 a	0.69	11.62 b	0.56
Water-soluble C (mg C kg ^−1^)	329.23 a	29.00	1710.35 c	204.32	601.25 b	45.88
Respiration (mg CO_2_-C kg^−1^ soil d^−1^)	22.80 a	2.48	32.45 b	3.65	37.28 b	1.58
β-glucosidase (μmol PNP g^−1^ h^−1^)	5.57 a	0.88	9.98 b	1.24	13.63 c	0.33
Cellulase (μg glucose g^−1^ h^−1^)	197.25 a	25.14	250.35 b	17.87	417.02 c	59.23
Lipase (μmol PNP g^−1^ min^−1^)	0.96 a	0.06	1.35 b	0.24	2.35 c	0.20
Phosphatase (μmol PNP g^−1^ h^−1^)	4.50 a	0.32	7.05 b	0.51	10.95 c	0.98
Polyphenol oxidase (mmol pyrogallol g^−1^ h^−1^)	114.90 a	8.59	146.36 b	24.67	154.64 b	12.80
Urease (μmol NH_4_^+^ g^−1^ h^−1^)	1.61 a	0.13	2.23 b	0.24	2.95 c	0.28

Data followed by the same letter are not significantly different according to the Tukey post-hoc test (HSD, *P* < 0.05).

**Table 2:** The abundance of bacterial and fungal proteins, diversity indexes and ratios between microbial proteins and energy proteins.

	Control		Soil + Sludge		Soil + Compost	
	*Mean*	*Std*^7^	*Mean*	*Std*	*Mean*	*Std*
Bacteria (%)	88.02 a	1.44	95.45 b	1.89	95.38 b	1.87
Fungi (%)	11.98 b	1.40	4.55 a	1.37	4.62 a	1.15
Ascomycota (%)	93.73 b	0.81	85.72 a	1.90	95.95 b	7.02
Basidiomycota (%)	6.27 b	0.85	14.28 c	0.78	4.05 a	0.47
Fungi/Bacteria	0.14 b	0.02	0.05 a	0.01	0.05 a	0.02
Shannon-B^1^	1.85 c	0.01	1.77 b	0.03	1.62 a	0.02
Simpson-B^2^	4.01 c	0.08	3.56 b	0.07	3.11 a	0.13
Shannon-F^3^	2.32 c	0,07	1.59 b	0.07	1.86 a	0.12
Simpson-F^4^	6.98 b	1.09	3.62 a	0.47	4.38 a	0.87
BG-En^5^	9.60 a	0.75	9.03 a	0.76	11.78 b	1.37
FG-En^6^	7.14 a	0.99	13.93 b	3.42	10.58 b	2.33

^1^The Shannon index of bacterial diversity.

^2^The Simpson index of bacterial diversity.

^3^The Shannon index of fungal diversity.

^4^The Simpson index of fungal diversity.

^5^Ratio between the total bacterial proteins and bacterial proteins involved in energy production.

^6^Ratio between the total fungal proteins and fungal proteins involved in energy production.

^7^Standard deviation of the mean. Data followed by the same letter are not significantly different according to the Tukey post-hoc test (HSD, *P* < 0.05).
