# Supplementary material for: Soil restoration with organic amendments: linking cellular functionality and ecosystem processes
Source: Sci Rep. 2015 Oct 27;5:15550. doi: 10.1038/srep15550 (PMC4621494; doi:10.1038/srep15550)
Supplement: Supplementary Information [file srep15550-s1.doc]

**Supplementary Information**

**Soil restoration with organic amendments: linking cellular functionality and ecosystem processes**

*F. Bastida^1*^, N. Selevsek^2^, I.F. Torres^1^, T. Hernández^1^, C. García^1^*

**Material and Methods**

*Study area and experimental design*

The experimental plots were located in Murcia (southeast Spain) in an area greatly affected by soil degradation processes. The climate is semiarid Mediterranean. The mean annual rainfall is 300 mm, and rainfall distribution throughout the year is very irregular with two maxima (in October and April). The mean annual temperature is 17 ºC. The studied soil has a sandy clay loam texture and is classified as an Aridic calcisol^1^. The vegetation of the study area is open Mediterranean scrub with species such as *Asphodelus fistulosus*, *Salsola genistoides*, *Piptatherum milliaceum*, *Dactylis* sp., *Rosmarinus officinalis* and *Stipa capensis*.

On March 25, 2004, nine 4 m x 5 m plots were randomly established in an experimental area (38º1’N 1º12’q W) located in Murcia (southeast Spain). Each plot was separated from the others by a corridor (2 m wide). This area was almost bare, without vegetation, and was abandoned 10 years before, in 1994. Soil abandonment, together with the inadequate climate conditions and the lost of plant cover, are considered as the main starters of soil degradation and desertification in arid areas. In order to fight against soil degradation, in three of the plots, sewage sludge from a water-treatment plant located in Murcia was added at a rate of 12 kg m^-2^. The sludge was anaerobically digested for stabilization and hygienization. Compost made from the same material and with straw as bulking material was added to another three plots (CM) at 12 kg m^-2^. The composting process was carried out as follows: piles of about 3 m^3^ of sludge mixed with cereal straw at a 3:1 (v:v) ratio were prepared and turned periodically (every 4-5 days) for 3 months to maintain adequate aeration and to homogenize the composting mass. The composting mass passed through a thermophilic phase. The moisture content was maintained at about 60-70% of water holding capacity throughout the composting period. The remaining three plots received no amendment and acted as control. Compost and sewage sludge were incorporated into the top 15 cm of the soil using a rotovator. Control plots were also subjected to rotovator treatment. The heavy metal contents in both the sludge and compost were below the limits established by the EU (Directive 86/278(CEE) for sludges and soils. The plots were left in natural conditions.

After 10 years, in April-2014, the plots were sampled. The samples were sieved at < 2 mm and stored at 4ºC until biochemical analysis and at -20ºC until proteomic analysis. Before sieving, all debris and plant remains were removed in order to avoid any influence on the parameters analyzed. For each sampling, 8 subsamples per plot were randomly collected from the upper 15 cm of soil surface with hand driven probes (10 cm diameter) and then mixed to constitute a single sample per plot. All samples were taken in triplicate. Analytical characteristics of the organic amendment are presented in Supporting Information (Table S1). Plant cover was higher in restored plots (81% in compost-amended plots and 85% in sludge-amended plots) than in control plots (42%), indicative of successful soil restoration.

**Table S1.** Characteristics of the different organic materials applied to soil.

|  | ***Sludge*** | ***Compost*** |
| --- | --- | --- |
| ***Parameter*** |  |  |
| **pH** | 6.6 | 6.8 |
| **Electrical conductivity (25ºC, 1:5)** ^1^**,** dS m^-1^ | 2.85 | 2.46 |
| **Total Organic C,** g kg^-1^ | 378 | 406 |
| **Total N,** g kg^-1^ | 43.0 | 30.3 |
| **Total P,** mg kg^-1^ | 1600 | 1700 |
| **Total K ,** mg kg^-1^ | 3900 | 4000 |
| **Total Cd,** mg kg^-1^ | < 2.5 | < 2.5 |
| **Total Ni,** mg kg^-1^ | 14.8 | 11.9 |
| **Total Cr,** mg kg^-1^ | 14.0 | 11.0 |
| **Total Zinc,** mg kg^-1^ | 718 | 510 |
| **Total Cu,** mg kg^-1^ | 247 | 187 |
| **Total Pb,** mg kg^-1^ | 80.8 | 54.9 |
| **C/N Ratio** | 8.73 | 13.5 |

*Chemical analyses, basal respiration and enzyme activities*

The pH was measured in a 1/5 (w/v) aqueous solution in a pH meter (Crison mod.2001, Barcelona, Spain). Total organic carbon (TOC) was determined using a C analyzer (Thermo Finnigan Flash EA 1112). Water-soluble carbon (WSC) was determined through soil extraction (2 h shaking with a soil:distilled water ratio of 1:5), followed by centrifugation, filtration, and analysis of the extract solution on a C analyzer for liquid samples (Shimazdu 5050A).

Microbial respiration (CO_2_ emission) was measured in 10 ml capped tubes containing 1 g of soil. Soil samples were humified with distilled water at 60% of their water-holding capacity. Vials were hermetically closed and incubated in the dark at 28 ºC for 11 days. The concentration of CO_2_ was periodically analyzed with a gas chromatograph [Trace Ultra Thermo Scientific, Milan (Italy)] using a packed column [Trace PLOT TG-BOND Q GC, Trace Ultra Thermo Scientific, Milan (Italy)].

The urease activity in the soil was determined by the buffered method of Kandeler & Gerber (1988)^2^. Phosphomonoesterase and β-glucosidase activities were determined following the methods described by Tabatabai & Bremner (1969)^3^ and a modification of Tabatabai’s method (1982)^4^, respectively. Polyphenol oxidase was determined by the method of Allison (2006)^5^ using 50 mM pyrogallol/50 mM EDTA as the substrate, and the absorbance of the supernatant from the reaction was determined directly at 460 nm after 1 h. Lipase was measured according to the method of Margesin et al. (2002)^6^ by reaction with p-nitrophenyl butyrate 100 mM. Cellulase activity was determined following a modification of the method of Deng & Tabatabai (1994)^7^ using carboxymethylcellulose as substrate.

*Phospholipid fatty acid analysis (PLFA)*

Phospholipids were extracted from 6 g of soil using chloroform-methanol extraction as described by Bligh & Dyer (1959)^8^ and fractionated and quantified using the procedure described by Frostegard et al. (1993)^9^. Phospholipids were transformed into fatty acid methyl esters (FAMEs) by alkaline methanolysis^10^ and designated as described by Frostegard et al. (1993)^9^. The complete dried FAME fraction was dissolved in isooctane containing 0.23 mg ml^-1^ of 21:0 FAME as internal standard. The analysis was perfomed using a Trace Ultra Thermo Scientific gas chromatograph fitted with a 60 m capillary column (Thermo TR-FAME 60m x 0.25 mm ID x 0.25 µm film), using helium as carrier gas.

The following fatty acids are characteristic bacterial fatty acids and were chosen as bacterial biomarkers: i15:0, a15:0, 15:0, i16:0, i17:0, cy17:0, cy19:0, 16:1ω7c, 16:1ω7t, 18:1ω9c and 18:1ω9t ^9,11^. The fatty acid 18:2ω6 was used as indicator of fungal biomass^12,13^. The Gram-positive representative fatty acids used were i15:0, a15:0, i16:0, and i17:0. The Gram-negative fatty acids used were cy17:0, cy19:0, 16:1ω7c, 16:1ω7t, 18:1ω9c and 18:1ω9t ^9,11^.

*Protein extraction and amino acid quantification of protein pellets*

Protein extraction was done according to the method described by Chourey et al*.* (2010)^14^, which was tested beforehand for semiarid soils^15^. The cell lysis and disruption of soil aggregates was performed by boiling for 10 minutes in SDS-buffer. The concentration and purification steps were performed using trichloroacetic acid (TCA) and three acetone washing steps.

The protein pellets resulting from each extraction protocol were dissolved in 50 µl of bi-distilled water, and 50 µl of 200 mM HCl were added for acid hydrolysis. The whole sample (100 µl) was hydrolyzed at 110°C for 24 h. The pH was equilibrated by the addition of borate-NaOH buffer. Twenty-five microlitres of each protein pellet lysate were derivatized according to the manufacturer´s instructions (Waters). One microliter was injected for amino acid analysis in a Waters Acquity Ultra Performance Liquid Chromatography (UPLC) System equipped with an AccQ-Tag pre-column derivatization UV detector of amino acid derivatives. Norvaline was used as the internal standard. The total amount of amino acids obtained after acid hydrolysis of the protein pellets was taken as the protein content and served for the standardization of the downstream proteomic pipeline.

*Sample preparation for proteomic analysis*

Protein pellets were resuspended in sodium dodecyl sulfate (SDS) lysis buffer containing 4% SDS, 0.1 mM dithiothreitol and 100 mM Tris HCl and incubated for 5 min at 95°C. High-intensity focused ultrasound (HIFU) was performed to solubilize proteins. Protein extracts were then subjected to ultrafiltration for detergent removal, cysteine alkylation and protein digestion according to Wisniewski et al. (2009)^16^. In the first step, SDS-containing buffer was replaced by urea buffer using several centrifugation steps. A filter device with relative molecular mass (Mr) cut-offs of 30000 (30k filter) was used to efficiently retain proteins and allow removal of impurities. Briefly, 200 µl of urea in 100mM Tris/HCl pH 8.2 were added, and the samples were centrifuged at 14000g for 20 min at room temperature. This step was repeated once. Then 100 µl of 0.05 M iodacetamide was added to the filters and centrifuged at 14000g for 20 min at room temperature. Filters were washed three times with urea buffer and followed by two washes with 0.5 M NaCl. Proteins were digested in 120 µl of TEAB using trypsin at an enzyme to protein ratio of 1:50 in a wet-cell chamber for 18 hours. The peptides were collected by centrifugation at 14000g for 20 min, and digestion was stopped by adding formic acid to a final concentration of 1%. Peptide mixtures were desalted using Finisterre SPE C18 reverse phase cartridges (Wicom International AG, Maienfeld, Germany) according to the following procedure: wetting the cartridge with 1 volume of 100% methanol, washing with 1 volume of 80% acetonitrile, equilibrating with 4 volumes of 0.1% formic acid, loading acidified digest, washing 6 volumes of 0.1% formic acid, and eluting with 1 volume of 50% acetonitrile in 0.1% formic acid. Peptides were dried using a vacuum centrifuge and resolubilized in 0.1% formic acid and frozen at -20°C.

*Mass spectrometric analysis of protein extracted from soils*

Tryptic peptides were analyzed on a Q Exactive mass spectrometer (Thermo Fisher Scientific, Bremen, Germany). Chromatographic separation of peptides was performed on an Easy nano-flow HPLC system (Thermo Fisher Scientific, Odense, Denmark) coupled to a 15 cm fused silica emitter, 75 μm in diameter, packed with a ReproSil-Pur C18-AQ 120 A and 1.9 μm resin (Dr. Maisch HPLC GmbH, Ammerbuch-Entringen, Germany). Peptides were loaded on the column and separated with a linear gradient of acetonitrile/water, containing 0.1% formic acid, at a flow rate of 300 nl/min. A gradient of 2 to 35 % acetonitrile in 120 min was used. Mass spectra were acquired in a data-dependent manner, with an automatic switch between MS and MS/MS using a top 12 method. MS spectra were acquired in the Orbitrap analyzer with a mass range of 300–1700 m/z and 70000 resolution at m/z 200. HCD peptide fragments, acquired at 28 normalized collision energy, were analyzed at high resolution in the Orbitrap.

*Database searching and bio-informatic classification of protein groups*

Each file was searched with Mascot (version 2.4.1) against the NCBInr database (08/24/2014, containing 48.094.830 sequences; 17.186.091.396 residues). For the search, one missed cleavage per peptide was allowed, and we used carbamidomethylation as a fixed modification on cysteine and oxidation as a variable modification on methionine residues. Searches were performed with a parent-ion mass tolerance of ±10 ppm and a fragment-ion mass tolerance of ±0.05 Da. The Mascot search results were imported to Scaffold (version Scaffold 4, Proteome Software, Portland, OR, USA) to validate MS/MS-based peptide and protein identifications. Proteins were considered to be identified with a protein threshold of 90% and a minimum of 2 peptides. Peptides were considered to be identified by FDR <1.3%.

The “PROteomics results Pruning & Homology group ANotation Engine” (PROPHANE)^17^ (<http://www.prophane.de/index.php?p=new>) was used to assign proteins to their phylogenetic (Figure 1) and functional (Figure 2) origin. Protein abundances were calculated based on the normalized spectral abundance factor (NSAF)^18^. Shannon and Simpson indexes were utilized for the calculation of the diversity of bacterial (phylum-level) and fungal (order-level) communities.

*Statistic analysis*

Statistical analyses were performed using IBM-SPSS Statistics (version 19.0) software. In order to determine pair-wise differences between the treatments, the data were analyzed using one-way ANOVA followed by the Tukey post-hoc test (HSD, *P*<0.05). The structure of the microbial community was visualized using multivariate factor analysis,with the relative abundances of phyla based on proteomics and the relative abundance of protein functional groups.

**References**

1. FAO-ISRIC-ISSS. *World Reference Base for Soil Resources*. FAO, Rome, 145 (1998).
2. Kandeler, E. & Gerber, H. Short-term assay of soil urease activity using colorimetric determination of ammonium. *Biol. Fertil. Soils* **6,** 68-72 (1988).
3. Tabatabai, M.A. & Bremmer, J.M. Use of p-nitrophenyl phosphate for assay of soil phosphate activity. *Soil Biol. Biochem.* **1,** 301-307 (1969).
4. Tabatabai, M.A. Soil Enzymes. In: Page, A.L., Miller, R.H., Keeney, D.R. (Eds.), Methods of Soil Analysis, Part 2. Chemical and Microbiological Properties, 2nd. Edition. American Society of Agronomy-Soil Science Society of America, Madison, 903-947 (1982).
5. Allison, S. & Jastrow, J.D. Activities of extracellular enzymes in physically isolated fractions of restored grassland soils. *Soil Biol. Biochem.* **38,** 3245-3256 (2006).
6. Margesin, R., Feller, G., Hammerle, M., Stegner, U. & Schinner, F. A colorimetric method for the determination of lipase activity in soil. *Biotechnol. Lett.* **24,** 27-33 (2002).
7. Deng, S.P. & Tabatabai, M.A. Colorimetric determination of reducing sugars in soil. *Soil Biol. Biochem.* **26,** 473-477 (1994).
8. Bligh, E.G. & Dyer, W.J. A rapid method for total lipid extraction and purification. *Can. J. Biochem. Physiol.* **37,** 911-917 (1959).
9. Frostegard, A., Tunlid, A. & Baath, E. Phospholipid fatty-acid composition, biomass, and activity of microbial communities from 2 soils types experimentally exposed to different heavy-metals. *Appl. Environ. Microbiol.* **59,** 3606-3617 (1993).
10. Guckert, J.B., Antworth, C.P., Nichols, P.D. & White, D.C. Phospholipid, ester-linked fatty acids profiles as reproducible assays for changes in prokaryotic community structure of estuarine sediments. *FEMS Microb. Ecol.* **31,** 147-158 (1985).
11. Dungait, J.A.J. et al. Variable responses of the soil microbial biomass to trace concentrations of 13C-labelled glucose, using 13C-PLFA analysis. *Eur. J. Soil Sci.* **62,** 117-126 (2011).
12. Brant, J.B., Sulzman, E.W. & Myrold, D.D. Microbial community utilization of added carbon substrates in response to long-term carbon input manipulation. *Soil Biol. Biochem.* **38,** 2219-2232 (2006).
13. **Rinnan, R. & Bååth, E.** Differential utilization of carbon substrates by bacteria and fungi in tundra soil. [*Appl. Environ. Microb.* **75,** 3611-3620](http://dx.doi.org/10.1128/AEM.02865-08) (2009).
14. Chourey, K. *et al.* Direct cellular lysis/protein extraction protocol for soil metaproteomics. *J. Proteome Res.* **9,** 6615-6622 (2010).
15. Bastida, F., Hernández, T. & García, C. Metaproteomics of soils from semiarid environment: Functional and phylogenetic information obtained with different protein extraction methods. *J. Proteomics* **101,** 31-42 (2014).
16. Wisniewski, J., *et al.* Universal sample preparation method for proteome analysis. *Nat. Meth.* **6,** 359-362 (2009).
17. Schneider, T. *et al.* Structure and function of the symbiosis partners of the lung lichen (*Lobaria pulmonaria* L. Hoffm.) analyzed by metaproteomics. *Proteomics* **11,** 2752-6 (2011).
18. Zybailov, B. *et al.* Statistical analysis of membrane proteome expression changes in *Saccharomyces cerevisiae*. *J. Proteome Res.* **5,** 2339-2347 (2006).
